# Identification and Prediction of Novel Clinical Phenotypes for Intensive Care Patients With SARS-CoV-2 Pneumonia: An Observational Cohort Study

**DOI:** 10.3389/fmed.2021.681336

**Published:** 2021-06-04

**Authors:** Hui Chen, Zhu Zhu, Nan Su, Jun Wang, Jun Gu, Shu Lu, Li Zhang, Xuesong Chen, Lei Xu, Xiangrong Shao, Jiangtao Yin, Jinghui Yang, Baodi Sun, Yongsheng Li

**Affiliations:** ^1^Department of Critical Care Medicine, The First Affiliated Hospital of Soochow University, Soochow University, Suzhou, China; ^2^Department of General Surgery, The Affiliated Suzhou Science & Technology Town Hospital of Nanjing Medical University, Suzhou, China; ^3^Department of Respiratory and Critical Care Medicine, The First Affiliated Hospital of Soochow University, Soochow University, Suzhou, China; ^4^Department of Respiratory Medicine, Affiliated Hospital of Nantong University, Nantong, China; ^5^Department of Intensive Care Unit, Affiliated Hospital of Nantong University, Nantong, China; ^6^Department of Respiratory Medicine, Zhongda Hospital Southeast University, Nanjing, China; ^7^Department of Respiratory and Critical Care Medicine, The First Affiliated Hospital of Nanjing Medical University, Nanjing, China; ^8^Department of Emergency Medicine, The Affiliated Hospital of Xuzhou Medical University, Xuzhou, China; ^9^Department of Respiratory Medicine, The Affliliation Hospital of Yangzhou University, Yangzhou, China; ^10^Department of Intensive Care Unit, The Affiliated Hospital of Jiangsu University, Zhenjiang, China; ^11^Department of Critical Care Medicine, Sir Run Run Hospital, Nanjing Medical University, Nanjing, China; ^12^Department of Emergency, Sir Run Run Hospital, Nanjing Medical University, Nanjing, China; ^13^Department of Intensive Care Medicine, Tongji Medical College, Tongji Hospital, Huazhong University of Science and Technology, Wuhan, China

**Keywords:** COVID-19, phenotypes, machine learning, intensive care unit, 28-day mortality

## Abstract

**Background:** Phenotypes have been identified within heterogeneous disease, such as acute respiratory distress syndrome and sepsis, which are associated with important prognostic and therapeutic implications. The present study sought to assess whether phenotypes can be derived from intensive care patients with coronavirus disease 2019 (COVID-19), to assess the correlation with prognosis, and to develop a parsimonious model for phenotype identification.

**Methods:** Adult patients with COVID-19 from Tongji hospital between January 2020 and March 2020 were included. The consensus k means clustering and latent class analysis (LCA) were applied to identify phenotypes using 26 clinical variables. We then employed machine learning algorithms to select a maximum of five important classifier variables, which were further used to establish a nested logistic regression model for phenotype identification.

**Results:** Both consensus k means clustering and LCA showed that a two-phenotype model was the best fit for the present cohort (*N* = 504). A total of 182 patients (36.1%) were classified as hyperactive phenotype, who exhibited a higher 28-day mortality and higher rates of organ dysfunction than did those in hypoactive phenotype. The top five variables used to assign phenotypes were neutrophil-to-lymphocyte ratio (NLR), ratio of pulse oxygen saturation to the fractional concentration of oxygen in inspired air (Spo_2_/Fio_2_) ratio, lactate dehydrogenase (LDH), tumor necrosis factor α (TNF-α), and urea nitrogen. From the nested logistic models, three-variable (NLR, Spo_2_/Fio_2_ ratio, and LDH) and four-variable (three-variable plus TNF-α) models were adjudicated to be the best performing, with the area under the curve of 0.95 [95% confidence interval (CI) = 0.94–0.97] and 0.97 (95% CI = 0.96–0.98), respectively.

**Conclusion:** We identified two phenotypes within COVID-19, with different host responses and outcomes. The phenotypes can be accurately identified with parsimonious classifier models using three or four variables.

## Introduction

Severe acute respiratory syndrome coronavirus 2 (SARS-CoV-2) pneumonia is a newly recognized infectious disease first reported in Wuhan, China, and expeditiously spread to hundreds of countries with massive mortality rate (1–4). The clinical spectrum of coronavirus disease 2019 (COVID-19) ranges from asymptomatic infection to critical illness and results in high rates of hospitalization and intensive care unit (ICU) admission (5). However, COVID-19 ICU mortality was various (6–8), and the treatment responses were disparate (9–11), indicating that COVID-19 is clinically and biologically heterogeneous.

Various studies have proposed different phenotypes of COVID-19. According to 85 consecutive ICU COVID-19 patients, Azoulay et al. identified three clinical and biological phenotypes at ICU admission using hierarchical clustering. ICU mortality rates were 8, 18, and 39% in clusters 1, 2, and 3, respectively (12). Gattinoni et al. identified two primary phenotypes based on respiratory mechanics and response to ventilatory support (13). Rello et al. classified COVID-19 patients into five specific individual phenotypes, according to the disease severity and hypoxemia management strategy (14). Whereas these phenotypes were isolated and limited by sample size, host responses to SARS-CoV-2 infection were vast and multidimensional and include immune dysfunction, abnormal coagulation, and varying degrees of organ failure (15). Different combinations of these features may cluster into novel clinical phenotypes, and patients in each phenotype may respond differently to treatments. However, whether such COVID-19 phenotypes can be derived from clinical data have never been explored.

Unsupervised machine learning approaches, such as consensus k means clustering (16) and latent class analysis (LCA) (17), have been used to identify distinct phenotypes in sepsis (18), acute respiratory distress syndrome (ARDS) (19) and other critical illnesses (20). Consensus clustering is a partitioning approach in which the clustering framework incorporates results from multiple runs of an inner-loop clustering algorithm. LCA is a well-validated statistical technique, which is a form of distribution mixture modeling used to estimate the best-fitting model for a dataset, based on the hypothesis that the data contain several unobserved groups or classes that are concealed within the observed multivariate distribution. Here, we used consensus k means clustering to derive phenotypes and assessed the reproducibility of the phenotypes using LCA.

The first goal of the study was to identify novel clinical phenotypes in ICU COVID-19 patients, using consensus k means clustering and LCA. The second goal was to develop parsimonious models that could ultimately be used prospectively to identify COVID-19 phenotypes.

## Materials and Methods

### Study Design and Participants

This single-center, retrospective, observational study was performed at Tongji Hospital, which was designated to admit patients with SARS-CoV-2 infection in Wuhan. Adult patients (≥18 years) with laboratory-confirmed SARS-CoV-2 infection and admitted to ICUs between January 2020 and March 2020 were included in the present study. According to the World Health Organization guidance (21), laboratory confirmation for SARS-Cov-2 was defined as a positive result of real-time reverse transcriptase–polymerase chain reaction assay of nasal and pharyngeal swabs.

This study was approved by the Research Ethics Commission of Tongji Hospital. Written informed consent was waived by the Ethics Commission because of the emergency circumstance. Patient-level informed consent was not required. Part of present patients have been described previously by Chen et al. (22) and Wang et al. (23).

### Data Collection

All data were drawn from electronic health record data at Tongji hospital (Tongji cohort). Demographic data, chronic comorbidities, vital signs, and laboratory results within the first 24 h after ICU admission were collected, as well as treatments and outcomes. Because of incomplete measurement and recording of arterial oxygen partial pressure (PaO_2_), we adopted pulse oxygen saturation (Spo_2_) instead of PaO_2_, as well as the fraction of inspired oxygen (Fio_2_). Sequential Organ Failure Assessment (SOFA) scores were calculated to determine the severity of illness using data from the first 24 h of ICU admission. All patients were closely followed until 28 days after ICU admission. Data were collected using a case record form modified from the standardized International Severe Acute Respiratory and Emerging Infection Consort.

### Outcomes

The primary outcome in the present study was 28-day mortality. Secondary outcomes were the duration of hospital stay and complications during hospitalization, which included ARDS, septic shock, acute kidney injury, acute cardiac injury, and coagulopathy. The diagnosis of complications is presented in the Supplementary Material.

### Clinical Variables for Phenotyping

We selected 26 candidate clinical variables based on their association with severity or outcome of COVID-19, including age, vital signs (heart rate, respiratory rate, temperature, mean blood pressure), markers of inflammation [white blood cell count (WBC count), neutrophil-to-lymphocyte ratio (NLR), high-sensitivity C-reactive protein (hs-CRP), interleukin 2R (IL-2R), IL-6, IL-8, and tumor necrosis factor α (TNF-α)], markers of organ dysfunction [hypersensitive troponin I (hs-TnI), international normalized ratio (INR), platelet (PLT) count, total bilirubin, creatinine, urea nitrogen, lactate dehydrogenase (LDH), and Spo_2_/Fio_2_ ratio], hemoglobin, red blood cell distribution width (RDW), d-dimer, fibrinogen, albumin, and glucose. All variables were collected within 24 h of ICU admission, and we recorded the most abnormal value if a variable was recorded more than once.

### Consensus k Means Clustering

Consensus k means clustering was conducted to 26 variables using a partitioning approach. We first assessed the candidate variable distributions, missingness, and correlation. Multiple imputations with chained equations (Additional Methods in Supplementary Material) were used to account for missing data; standardized transformation was used for the dataset, and non–normally distributed variables were log-transformed prior to standardized transformation. We then determine the optimal number of phenotypes with consensus k means clustering, according to the gap statistics, consensus matrix heatmaps, and adequate pairwise-consensus values between cluster members (>0.8). Once the optimal number was determined, we selected rank plots of variables by mean standardized difference between phenotypes to visualize the patterns of clinical variables. We also conducted a sensitivity analysis after excluding highly correlated variables using rank-order statistics (*r* > 0.5). Additional details of consensus k means clustering are presented in Supplementary Material.

### Latent Class Analysis

We further employed LCA to assess the reproducibility of the phenotypes. Similarly, all variables underwent standardized transformation and were log-transformed as appropriate. In the LCA, we estimated models ranging from to five classes. Akaike information criterion (AIC), Bayesian information criteria, entropy, class size (classes containing relatively small numbers were not considered clinically meaningful), and the Vuong–Lo–Mendell–Rubin (VLMR) likelihood ratio test (which compares fit of model k classes to k-1 classes) were used to determine the optimal number of classes. Once determined, each individual was assigned a class according to model-generated probabilities. More details of LCA are presented in the Supplementary Material.

### Parsimonious Algorithms to Classify COVID-19

Based on previous research, we attempted to construct a parsimonious model (three-variable or four-variable model) to predict phenotypes. First, machine learning algorithms, including classification tree with bootstrapped aggregating (bagging), extreme gradient boosting (XGBoost), and gradient boosted model (GBM), were used to identify the most important classifier variables. To select the most important variables, variable importance was used for the bagging model and XGBoost. Relative influence factor of variable was used for GBM. More details of machine learning algorithms are presented in the Supplementary Material. Second, the five most important classifier variables common to all three machine learning algorithms were then used to generate five logistic regression models (generated by sequential addition of the variables), and the receiver operating characteristic curve and area under the curve (AUC) were calculated for each model. AIC and DeLong's test were used to compare model performance. The best model was determined by a combination of accuracy, parsimony, and simplicity in clinical. Additionally, to assess the clinical usefulness of the best model, decision curve analysis (DCA) was conducted by quantifying the net benefits at different threshold probabilities. Finally, after the best model selected, a 10-fold cross-validation was applied to internally validate the stability of the model. This was performed by randomly splitting the patients into 10 equal samples. Nine-tenths of these samples were used to construct logistic regression models, and the model coefficients were applied to the remaining sample (1/10). This process was repeated 10 times, and the AUC to each fold was generated.

### Statistical Analysis

Values are presented as the mean (standard deviation) or median (interquartile range) for continuous variables as appropriate and as the total number (percentage) for categorical variables. Comparisons between groups were made using the χ^2^ test or Fisher exact test for categorical variables and Student *t*-test or Mann–Whitney *U*-test for continuous variables as appropriate. A *p* < 0.05 was used to determine statistical significance for all tests. LCA was conducted using Mplus software (version 8.3). All other analyses were done using R (version 3.6.0).

## Results

### Patients

**Figure 1:**
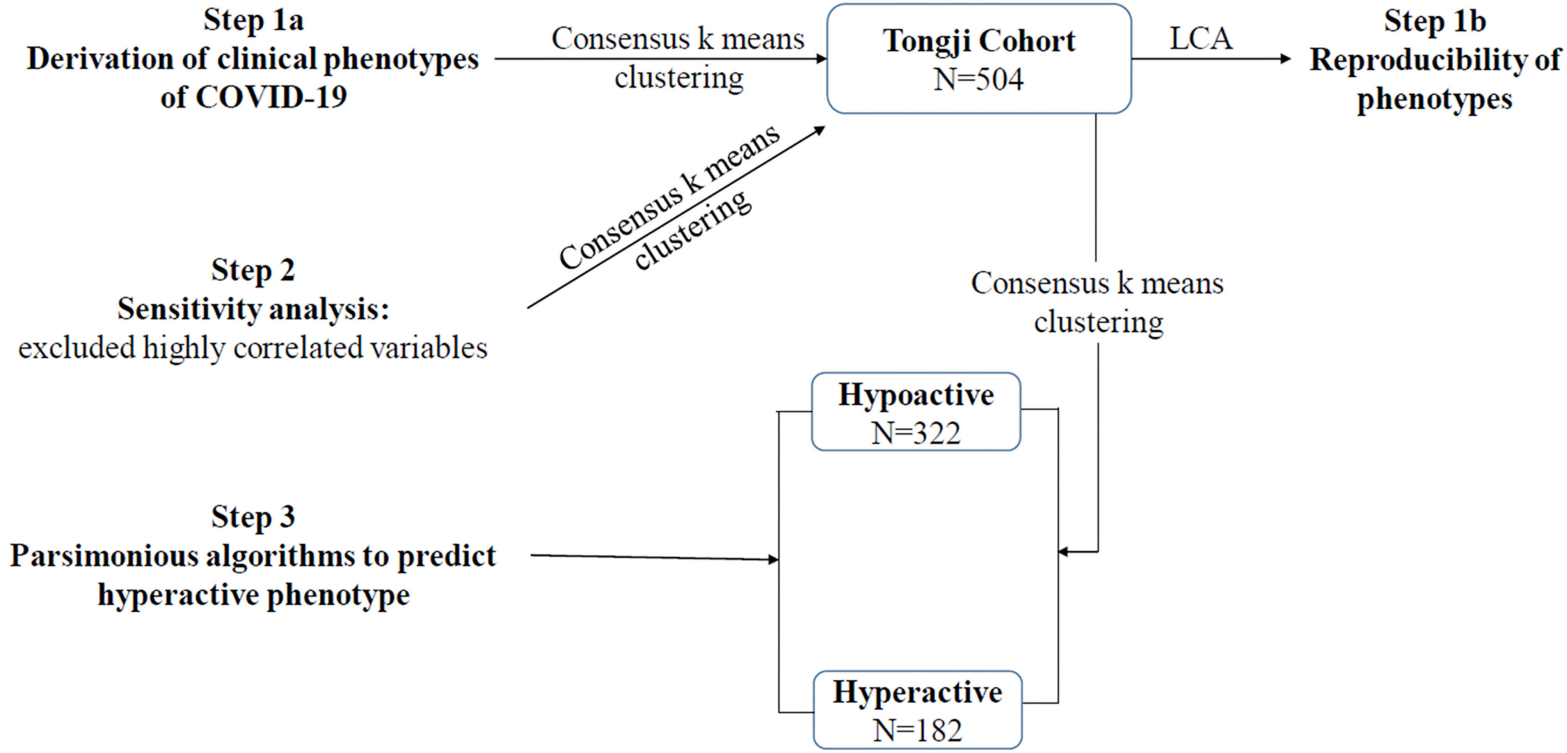
Schematic of study. LCA, latent class analysis.

During the study period, a total of 504 patients with COVID-19 were included in the Tongji cohort. The schematic of study is shown in Figure 1. Among the Tongji cohort, 259 patients (51.4%) were male, the age was 64 (52–72) years, and the SOFA score was 3 (2–6). Within the first 24 h after ICU admission, 16 patients (3.2%) received vasopressor therapy, and 23 patients (4.6%) received invasive mechanical ventilation. The overall 28-day mortality rate was 33.7%.

### Derivation of Clinical Phenotypes for COVID-19

In Tongji cohort, based on gap statistics, consensus matrix plots, and consensus values (Supplementary Figure 1), the consensus k means clustering found that a two-class model was the optimal fit with the two distinct phenotypes of COVID-19. Ultimately, 322 patients (63.9%) were classified as hypoactive phenotype, and 182 (36.1%) were classified as hyperactive phenotype. Sensitivity analysis indicated that no substantial changes were evident after excluding variables with high correlation (Supplementary Table 3 and Supplementary Figure 2).

**Figure 2:**
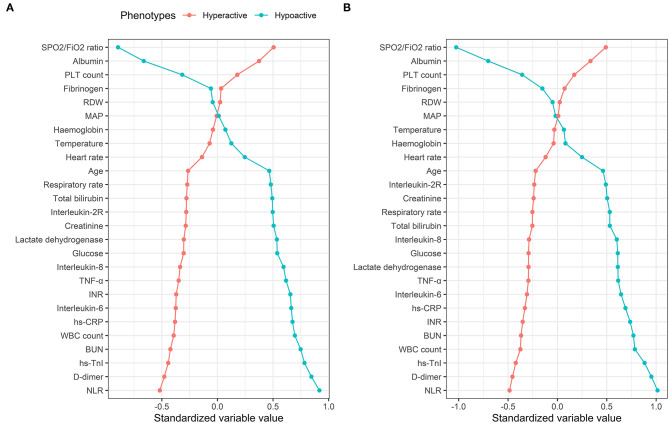
Comparison of variables that contribute to clinical phenotypes in the Tongji cohort. Clinical phenotypes were derived from consensus k means clustering **(A)** and LCA **(B)**. In all panels, the variables are standardized such that all means are scaled to 0 and SDs to 1. A value of 1 for the standardized variable value (*x*-axis) signifies that the mean value for the phenotype was 1 SD higher than the mean value for both phenotypes shown in the graph as a whole. RDW, red blood cell distribution width; MAP, mean arterial pressure; TNF-α, tumor necrosis factor α; INR, international normalized ratio; hs-CRP, high-sensitivity C-reactive protein; BUN, urea nitrogen; hs-TnI, hypersensitive troponin I; NLR, neutrophil-to-lymphocyte ratio.

The characteristics of phenotypes in the two-class model are shown in Table 1 and Supplementary Figure 3. Rank plots of variables by the standardized mean difference between phenotypes are presented in Figure 2. Most variables were significantly different between the two phenotypes. Compared to patients with the hypoactive phenotype, those with the hyperactive phenotype were older, prone to have elevated measures of inflammation (e.g., WBC count, NLR, hs-CRP, IL-2R, IL-6, IL-8, TNF-α), higher d-dimer, higher heart rate, higher respiratory rate, and extreme laboratory values regarding the organ dysfunction (e.g., hs-TnI, INR, PLT count, total bilirubin, creatinine, urea nitrogen, LDH, and Spo_2_/Fio_2_). Additionally, in comparison with the hypoactive phenotype, the hyperactive phenotype had significantly higher SOFA score on ICU admission and higher comorbidity rates (Supplementary Table 4).

**Table 1:** Class-defining variables of phenotypes using consensus k means clustering.

**Variables**	**Hypoactive phenotype (*n* = 322)**	**Hyperactive phenotype (*n* = 182)**	***p*-value**
Age (years)	58 (48–69)	69 (62–77)	<0.001
Heart rate (bpm)	89 (78–101)	95 (82–108)	<0.001
Respiratory rate (bpm)	20 (20–22)	24 (20–32)	<0.001
Temperature (°C)	37.0 (36.5–37.8)	37.2 (36.5–38.0)	0.063
MAP	96.0 (89.7–104.7)	99.7 (89.0–106.0)	0.209
Spo_2_/Fio_2_ ratio	297 (259–433)	131 (90–229)	<0.001
WBC count (× 10^9^/L)	5.2 (4.0–6.6)	9.4 (7.0–13.1)	<0.001
NLR	3.4 (2.0–5.4)	13.5 (8.6–25.3)	<0.001
Platelet count (× 10^9^/L)	213 (159–278)	164 (121–225)	<0.001
Hemoglobin (g/L)	126 (115–137)	129 (115–143)	0.043
RDW (%)	12.4 (11.9–13.2)	13.0 (12.2–13.9)	<0.001
High-sensitivity C-reactive protein (mg/L)	26.2 (5.6–65.2)	104.6 (65.0–163.4)	<0.001
Interleukin 2R (U/mL)	658 (426–906)	1,262 (904–1648)	<0.001
Interleukin 6 (pg/mL)	10.2 (2.3–31.1)	64.8 (31.0–157.0)	<0.001
Interleukin 8 (pg/mL)	11.4 (6.5–19.5)	32.3 (20.0–66.4)	<0.001
Tumor necrosis factor α (pg/mL)	7.8 (5.8–10.0)	12.8 (8.9–18.8)	<0.001
d–Dimer (μg/mL)	0.7 (0.4–1.4)	5.3 (1.8–21.0)	<0.001
Fibrinogen (g/L)	4.8 (4.0–5.9)	5.4 (3.3–6.5)	0.152
INR	1.0 (1.0–1.1)	1.2 (1.1–1.4)	<0.001
Hypersensitive troponin I (pg/mL)	3.8 (1.9–8.4)	40.1 (13.3–296.2)	<0.001
Albumin (g/L)	36.0 (33.3–38.6)	29.9 (27.1–32.7)	<0.001
Total bilirubin (μmol/L)	8.7 (6.5–11.7)	13.2 (9.9–19.2)	<0.001
Creatinine (μmol/L)	66.0 (55.8–82.0)	89.0 (71.5–119.0)	<0.001
Urea nitrogen (mmol/L)	4.2 (3.2–5.5)	9.3 (6.4–15.2)	<0.001
Lactate dehydrogenase (U/L)	260 (203–334)	511 (415–678)	<0.001
Glucose (mmol/L)	6.1 (5.2–7.2)	8.1 (6.3–11.8)	<0.001

*MAP, mean arterial pressure; Sp*o_2_/*F*io_2_ *ratio, ratio of pulse oxygen saturation to the fractional concentration of oxygen in inspired air; WBC, white blood cell count; NLR, neutrophil-to-lymphocyte ratio; RDW, red blood cell distribution width; INR, international normalized ratio*.

### Treatments and Outcomes in COVID-19 Phenotypes

A large proportion of patients with the hyperactive phenotype received corticosteroid therapy (78.6 vs. 44.1%; *p* < 0.001), high-flow nasal cannula oxygen therapy (17.0 vs. 4.7%; *p* < 0.001), non-invasive mechanical ventilation (45.6 vs. 7.1%; *p* < 0.001), invasive mechanical ventilation (59.3 vs. 3.4%; *p* < 0.001), and renal replacement therapy (11.5 vs. 1.6%; *p* < 0.001) during their ICU stay, compared to those with hypoactive phenotype (Supplementary Table 4). Patients assigned to hyperactive phenotype had significantly higher 28-day mortality (74.3 vs. 10.8%; *p* < 0.001) and higher rates of organ dysfunction during their ICU stay compared to those assigned to hypoactive phenotype (Table 2).

**Table 2:** Comparison of clinical outcomes according to phenotypes using consensus k means clustering.

	**Hypoactive phenotype (*n* = 322)**	**Hyperactive phenotype (*n* = 182)**	***p*-value**
ARDS	46 (14.3%)	149 (81.9%)	<0.001
Septic shock	25 (7.8%)	128 (70.3%)	<0.001
Coagulopathy	14 (4.3%)	84 (46.2%)	<0.001
Acute kidney injury	16 (5.0%)	96 (52.7%)	<0.001
Acute cardiac injury	32 (10.0%)	120 (65.9%)	<0.001
28-d mortality	35 (10.8%)	135 (74.3%)	<0.001

*ARDS, acute respiratory distress syndrome*.

### Reproducibility Using LCA

LCA confirmed statistical fit of the two-class model. In LCA, using the VLMR test, a two-class model showed significantly improved fit compared with one-class mode (*p* = 0.0066), and no further improvement in model fit was observed when the three-class (*p* = 0.058), four-class (*p* = 0.41), or five-class model (*p* = 0.40) was involved. Good class separation was observed in the two-class model (entropy > 0.80), indicating strong separation between the classes (Supplementary Table 5). The two-class model classified 341 patients (67.7%) in class 1 (referred as hypoactive phenotype) and 163 patients (32.3%) in class 2 (referred as hyperactive phenotype). Average latent class probabilities were 0.98 for class 1 and 0.96 for class 2. The clinical characteristics of the phenotypes were similar when derived using this method, as well as by rank plots (Figure 2 and Supplementary Table 6).

### Parsimonious Algorithms to Predict Phenotypes of COVID-19

The most important classifier variables from the bagging, XGBoost, and GBM are presented (Supplementary Table 7, Supplementary Figures 4, 5). The top five variables were consistent across all three machine learning models, which included NLR, Spo_2_/Fio_2_ ratio, LDH, TNF-α, and urea nitrogen, and were therefore selected as the best predictors for the parsimonious models. After five logistic models constructed by sequential addition of the best predictors, an improved model performance, increased AUC, and decreased AIC were observed when model 1 went to model 4 (Supplementary Table 8). Considering that TNF-α was not routinely tested in other hospitals, therefore, the three-variable (NLR, Spo_2_/Fio_2_ ratio, and LDH) and four-variable models (NLR, Spo_2_/Fio_2_ ratio, LDH, TNF-α) were both the best in terms of balancing classifying accuracy and model simplicity.

**Figure 3:**
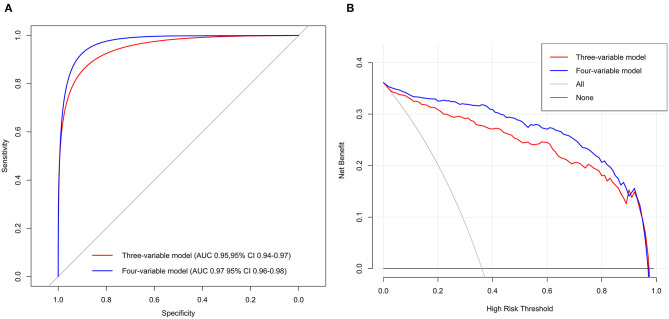
Receiver operating characteristic curves **(A)** and DCA **(B)** of the two best-performing regression models in Tongji cohort.

Multivariable analyses showed that three variables or four variables in the model were all predictors of the phenotypes (Supplementary Table 9). The AUC was 0.95 [95% confidence interval (95% CI) = 0.94–0.97] for the three-variable model and 0.97 (95% CI = 0.96–0.98) for the four-variable model. The DCA curves indicated that the threshold probabilities were 0–0.95 for the three-variable model and 0–0.94 for the four-variable model (Figure 3). The mean AUCs of cross-validation for the three- and four-variable models were 0.95 (0.03) and 0.97 (0.02), respectively.

## Discussion

The novel findings of our analyses can be summarized as follows. We identified two distinct COVID-19 phenotypes with different clinical and biological characteristics, mortality, and other clinical outcomes. We also developed a parsimonious model to predict phenotypes of COVID-19 using machine learning algorithms. These findings have important implications for early detection of patients who are likely to develop critical illness, as well as future researches in COVID-19.

Clinical and biological heterogeneity of critical illness (e.g., ARDS, sepsis) is thought to be dead ends for pharmacotherapy trials. Not a single clinical or biological variable was sufficient to identify phenotype (24). To put it simple, none of the clinical variables could be used to subdivide COVID-19. By contrast, based on 26 candidate clinical variables, we found two distinct phenotypes of COVID-19 most sufficiently describing the present cohort using consensus k means clustering, which strongly correlated with degrees of the host response to SARS-CoV-2 infection. Specifically, compared to patients with hypoactive phenotype, the host response of patients with hyperactive phenotype seems to be more dysregulated, characterized by high plasma concentrations of inflammatory biomarkers, extreme coagulation, and high proportion of organ failure or injury on ICU admission. Furthermore, replication of these findings using LCA substantiates the robustness of the two phenotypes in the present cohort.

Several phenotypes of COVID-19 have been documented, with the aim to receive “precision therapy.” Patients with COVID-19 pneumonia presents with low elastance, low ventilation-to-perfusion ratio, low lung weight, and low lung recruitability were classified as type L, whereas type H patients were characterized by high elastance, high ventilation-to-perfusion ratio, high lung weight, and high lung recruitability. Response to treatments, including higher Fio_2_ and higher positive end-expiratory pressure (PEEP), and prone positioning may differ in type L and type H (13). Compared to phenotypes in the present study, similarly, hyperactive phenotype and type H seemed to represent a subset of COVID-19 patients who were severely ill. Unlike previous COVID-19 phenotypes, the COVID-19 phenotypes in the present study only used routinely available data associated with the degrees of host response, regardless of the characteristics of chest imaging or the respiratory mechanics, which can be identified at the time of patient admitted to the ICU. Besides, these phenotypes were multidimensional, differed in their laboratory abnormalities, patterns of organ dysfunction, and were not homologous with traditional patient groupings such as by severity score or a single variable.

We proposed a three-variable (NLR, Spo_2_/Fio_2_ ratio, and LDH) and four-variable model (NLR, Spo_2_/Fio_2_ ratio, LDH, and TNF-α) for identifying the hyperactive phenotype of COVID-19. Unlike traditional forward stepwise modeling, we used three machine algorithms to identify the most important classifier variables. The ability to identify phenotypes using a small set of variables is a crucial step toward their clinical application. On the one hand, to predict the occurrence of critical illness in COVID-19: according to 1,590 COVID-19 patients, Wenhua Liang et al. (25) constructed a predictive risk score including 10 variables to predict a patient's risk of developing critical illness; likewise, NLR [odds ratio (OR) = 1.06; 95% CI = 1.02–1.10] and LDH (OR = 1.002; 95% CI = 1.001–1.004) were included in the risk model. However, the definition of “critical illness” was obscure, which was described as a composite of admission to the ICU, invasive ventilation, or death. Besides, the overall mortality was only 3.2%, implying that such risk score may not be validated in real intensive care patients with COVID-19. In the present study, the ICU mortality of Tongji cohort was in line with prior reports, and critically ill patients (hyperactive phenotype) were identified based on the clustering analysis and LCA, which maximized the differences between patients, without taking the clinical outcome into account (26). On the other hand, to select more homogeneity patients for clinical trials: hypothetically, like the series research of ARDS, the interactions between phenotypes and treatments (PEEP, fluid management, and simvastatin) were significant.

Interestingly, different from the ARDS phenotypes (24, 27), we observed that none of inflammatory cytokines could predict COVID-19 phenotypes, except for TNF-α. Proinflammatory cytokines levels (IL-6, IL-8) in hyperinflammatory ARDS were at least 20-fold higher than hyperactive COVID-19 in our study, suggesting that COVID-19 is associated with only mild inflammatory cytokine elevation. An alternative mechanism of disease therefore seems likely (28) and warrants further researches. Additionally, pulmonary-specific variables, such as PaO_2_/Fio_2_ ratio, seem to contribute less to phenotype identification in ARDS; nevertheless, Spo_2_/Fio_2_ ratio is a primary variable to classify COVID-19 phenotype in the present study. A potential explanation for this finding is that patients were enrolled into ARDS clinical trials based on specific pulmonary criteria (e.g., PaO_2_/Fio_2_ ratio), but COVID-19 patients in Tongji cohort are more heterogeneous with respect to pulmonary variables (e.g., Spo_2_/Fio_2_ ratio).

The first strength of our study is the identification of two class phenotypes for intensive care patients with COVID-19 and development of the first parsimonious model for predicting hyperactive phenotype. The observational nature of the present study is another strength as it included all consecutive patients with COVID-19 during 3 months, and the results are therefore more likely to represent the population as encountered in the ICU in clinical practice.

This study also has several limitations. First, our study is a single-center, retrospective, observational study, and we lack the external validation of the phenotypes and the parsimonious model. Testing for COVID-19 phenotypes in more heterogeneous samples is an important direction in future researches. Second, the 26 candidate clinical variables did not fully reflect the host response to SARS-CoV-2 infection; we cannot exclude that adding other markers would provide different phenotypes. Third, whether these phenotypes are dynamic and change over time, resulting in distinct COVID-19 trajectories, is unknown. Finally, although a three- or four-variable model has a good accuracy in predicting the phenotypes, when phenotypes are defined by the parsimonious model rather than the clustering analysis or LCA, we may no longer detect the statistically significant differences in outcomes and treatment responses.

## Conclusion

In summary, this analysis confirmed the existence of two distinct phenotypes for intensive care patients with COVID-19. We also provide evidence for accurate parsimonious classifier models of COVID-19 phenotypes. Promisingly, these simple models may aid clinicians in predicting which COVID-19 patients are likely to develop critical illness, delivering timely treatments, and improving patient selection in clinical trials, which in turn could significantly impact patient outcomes.

## Data Availability Statement

The original contributions presented in the study are included in the article/Supplementary Material, further inquiries can be directed to the corresponding author/s.

## Ethics Statement

The studies involving human participants were reviewed and approved by Tongji Hospital Ethics Committee. Written informed consent for participation was not required for this study in accordance with the national legislation and the institutional requirements.

## Author Contributions

YL and HC conceptualized the research aims, design the study, take responsibility for the integrity of the data and the accuracy of the data analysis. HC did the statistical analysis. HC and ZZ wrote the first draft of the manuscript. All authors contributed to acquisition of data, provided comments and approved the final manuscript.

## Conflict of Interest

The authors declare that the research was conducted in the absence of any commercial or financial relationships that could be construed as a potential conflict of interest.
