# Supplementary material for: Identification and Prediction of Novel Clinical Phenotypes for Intensive Care Patients With SARS-CoV-2 Pneumonia: An Observational Cohort Study
Source: Front Med (Lausanne). 2021 Jun 4;8:681336. doi: 10.3389/fmed.2021.681336 (PMC8211883; doi:10.3389/fmed.2021.681336)
Supplement: Supplementary file 1 [file Data_Sheet_1.docx]

**Identification and prediction of novel clinical phenotypes for intensive care patients with SARS-CoV-2 pneumonia: An observational cohort study**

Online Supplement

**Additional Methods**

A. Outcomes

B. Approach to missing data

C. Consensus K means clustering

D. Latent class analysis

E. Classification Tree with Bagging

F. Extreme gradient boosting

G. Gradient boosted model

**Additional References**

**Supplementary Table 1-** Percentage of missing data of class-defining variables in Tongji cohort

**Supplementary Table 2-** Class-defining variables before and after multiple imputation

**Supplementary Table 3-** Class-defining variables by phenotypes in sensitivity analysis excluding variables with high correlation (WBC count, Interleukin-6 and D-dimer)

**Supplementary Table 4-**Additional baseline characteristics and treatments of phenotypes using consensus K means clustering

**Supplementary Table 5-** Fit statistics for latent class models from one to five classes

**Supplementary Table 6-** Class-defining variables of phenotypes derived using latent class analysis

**Supplementary Table 7-** The importance of top five variables from the classification tree with bagging model

**Supplementary Table 8-** Nested model composition and accuracy in dataset

**Supplementary Table 9-** Multivariable models assessing impact of variables on classification of phenotypes

**Supplementary Figure 1-**Consensus k clustering results in Tongji cohort

**Supplementary Figure 2-**Heatmap of correlation between clinical variables for phenotyping

**Supplementary Figure 3-**Difference of key class-defining variables between phenotypes

**Supplementary Figure 4-**Feature importance derived from XGBoost model

**Supplementary Figure 5-**Relative influence of variables derived from GBM (A) and training process of GBM (B)

**ADDITIONAL METHODS**

**A. Outcomes**

ARDS was diagnosed based on the Berlin Definition [1], considered that we used SPO_2_ instead of PaO_2_, the oxygenation diagnostic criterion was changed to SPO_2_/FiO_2_ < 357 [2]. Septic shock was defined according to the 2016 Third International Consensus Definition for Sepsis and Septic Shock [3]. Acute kidney injury was diagnosed according to the KDIGO clinical practice guidelines [4]. Acute cardiac injury was diagnosed if serum levels of cardiac biomarkers (hypersensitive troponin I) were above the 99th percentile upper reference limit. Coagulopathy was defined as a 3-second extension of prothrombin time or a 10-second extension of activated partial thromboplastin time.

**B. Approach to missing data**

Missing data of class-defining variables were summarized in Supplementary Table 1. The percentage of missing data of variables were less than 20%, except for hypersensitive troponin I (20.2%). Prior to clustering algorithms, LCA and machine learning algorithms (Bagging, XGBoost and GBM), we assumed that missing data was “missing at random”, We used multiple imputation by chained equation (MICE) which generated values for all missing data using the observed data for all patients. In total we imputed 5 different data sets (n = 5), the imputation method was weighted predictive mean matching. We evaluated distributions of class-defining variables before and after imputation (Supplementary Table 2), and no differences were detected. We used ‘mice’ package in R studio to impute the data (https://cran.rproject.org/web/packages/mice).

**C. Consensus K means clustering**

Consensus clustering is a partitioning approach in which the clustering framework incorporates results from multiple runs of an inner-loop clustering algorithm (such as K-means, model-based Bayesian clustering, self-organizing map) on sub-sampled subjects. In present study, we chose K-means as the inner-loop clustering algorithm. There are three steps to conduct the consensus K means clustering. First, after multiple imputation, we standardized (minus mean and divided by standard deviation, giving a mean of 0 and standard deviation of 1.0) and normalized (log-transformed to obtain normally distributed variables) the dataset. Second, based on the combination of gap statistics, consensus matrix heatmaps, consensus values between cluster members, class size and clinical characteristics, we determined an optimal number of clusters. Specifically, i.) maximum gap statistics indicate the optimal estimate of the number of cluster in the data; ii.) for each given number of clusters, a consensus matrix is created as a heatmap, by varying numbers of clusters and comparing the heat maps, visual assessment can determine fit to the data; iii.) consensus value ranges from 0 to 1, with larger values indicating stronger consensus. A value of 0 means that the pair has never been assigned to the same cluster among times they are both sampled, whereas a value of 1 means that this pair has always been assigned to the same cluster among times they are both sampled, a number of clusters with higher cluster consensus (above 0.8) for all clusters is preferred. Finally, we selected rank plots of variables by the mean standardized difference between phenotypes to visualize the patterns of clinical variables, a value of 1 for the standardized variable value signifies that the mean value for the phenotype was 1 SD higher than the mean value for all patients.

**D. Latent class analysis (LCA)**

LCA is a well-validated statistical technique that is a form of distribution mixture modelling to estimate the best fitting model for a set of data, based on the hypothesis that the data contain several unobserved groups or classes which are concealed within the observed multivariate distribution. First, data process was as same as consensus k means clustering. Five separate models consisting of one, two, three, four and five classes were built, based on these five models, we then determined the optimal number of clusters (k) using a combination of criteria, i.) a smaller Akaike information criterion and Bayesian information criterion; ii.) adequate sample size within cluster; iii.) higher entropy, entropy ranges from 0 to 1, for a given subject and a given cluster, the higher the entropy is, the more likely this subject belongs to this cluster; iiii.) Vuong-Lo-Mendell-Rubin likehood ratio test (which compares fit of model k classes to k-1 classes) and clinical characteristics. Finally, we also selected rank plots described above to visualize the patterns of clinical variables in LCA.

**E. Classification Tree with Bagging**

Bagging is a widely used ensemble method for classification which combines bootstrapping and aggregating. If the bootstrap estimate of the data distribution parameters is more accurate and robust than the traditional one, then a similar method can be used to achieve, after combining them, the accuracy of classification is improving. The bagging algorithm was used to determine the most important variables in our study. Classification tree analysis and bagging were analysed using R Studio with the ‘adabage’ package (<https://cran.r-project.org/web/packages/adabage>).

**F. Extreme gradient boosting (XGBoost)**

XGBoost combined with decision trees was employed to predict phenotypes. A classification tree was used as the weak learner, and the learning objective function was binary logistic. The boosting method works by iteratively refitting a weak classifier (decision tree) to residuals of previous models. Each successive classifier focused more on misclassified observations during the previous round of fitting. In our study, in order to identify the most important classifier variables, the importance of classifier variables were derived from XGBoost model (learning rate = 0.1, minimum loss reduction = 0, maximum tree depth =20, and number of trees = 250). We used ‘xgboost’ package (<https://cran.rproject.org/web/packages/xgboost>) in R studio to impute the data.

**G. Gradient boosted model (GBM)**

GBM is a machine learning algorithm that consecutively constructs new models and forms an ensemble of models to provide a more accurate estimate of the response variable. The principal idea is to construct the new base-learners to be maximally correlated with the negative gradient of the predefined loss function. In our study, regression tree was used as the base learner of the GBM. The initial settings included a bag fraction of 0.5, a tree complexity of 8, the number of tree was 1128, and a learning rate of 0.01. We used GBM to generate the relative influence of class-defining variables. We used ‘gbm’ package (<https://cran.rproject.org/web/packages/gbm>) in R studio to impute the data.

**Additional References**

1. Ranieri VM, Rubenfeld GD, Thompson BT *et al* (2012) **Acute respiratory distress syndrome: the Berlin Definition**.*Jama* **307**(23):2526-2533. <https://doi.org/10.1001/jama.2012.5669>

2. Pandharipande PP, Shintani AK, Hagerman HE *et al* (2009) **Derivation and validation of Spo2/Fio2 ratio to impute for Pao2/Fio2 ratio in the respiratory component of the Sequential Organ Failure Assessment score**.*Critical care medicine* **37**(4):1317-1321. <https://doi.org/10.1097/CCM.0b013e31819cefa9>

3. Singer M, Deutschman CS, Seymour CW *et al* (2016) **The Third International Consensus Definitions for Sepsis and Septic Shock (Sepsis-3)**.*Jama* **315**(8):801-810. <https://doi.org/10.1001/jama.2016.0287>

4. Khwaja A (2012) **KDIGO clinical practice guidelines for acute kidney injury**.*Nephron Clinical practice* **120**(4):c179-184. <https://doi.org/10.1159/000339789>

**Supplementary Table 1: Percentage of missing data of class-defining variables in Tongji cohort.**

| Variables | Missing, n (%) |
| --- | --- |
| Age (years) | 0 (0%) |
| Heart rate (bpm) | 23 (4.6%) |
| Respiratory rate (bpm) | 28 (5.6%) |
| Temperature (℃) | 10 (2.0%) |
| MAP | 34 (6.7%) |
| SPO_2_/FiO_2_ ratio | 9 (1.8%) |
| WBC count (×10^9^/L) | 8 (1.6%) |
| NLR | 7 (1.4%) |
| PLT count (×10^9^/L) | 10 (2.0%) |
| Haemoglobin (g/L) | 7 (1.5%) |
| RDW (%) | 8 (1.6%) |
| High-sensitive C-reactive protein (mg/L) | 24 (4.8%) |
| Interleukin-2R (U/ml) | 95 (18.8%) |
| Interleukin-6 (pg/ml) | 94 (18.7%) |
| Interleukin-8 (pg/ml) | 95 (18.8%) |
| Tumour necrosis factor-α (pg/ml) | 96 (19.0%) |
| D-dimer (μg/ml) | 45 (8.9%) |
| Fibrinogen (g/L) | 65 (12.9%) |
| INR | 39 (7.7%) |
| Hypersensitive troponin I (pg/ml) | 102 (20.2%) |
| Albumin (g/L) | 5 (1.0%) |
| Total bilirubin (μmol/L) | 5 (1.0%) |
| Creatinine (μoml/L) | 5 (1.0%) |
| Urea nitrogen (mmol/L) | 5 (1.0%) |
| Lactate dehydrogenase (U/L) | 10 (2.0%) |
| Glucose (mmol/L) | 26 (5.2%) |

MAP= mean arterial pressure; SPO_2_/FiO_2_ ratio= ratio of pulse oxygen saturation to the fractional concentration of oxygen in inspired air; WBC= White blood cell count; NLR= Neutrophil-to-Lymphocyte ratio; RDW= Red blood cell Distribution Width; INR=International Normalized Ratio.

**Supplementary Table 2: Class-defining variables before and after multiple imputation**

| Variables | Observed cohort | Imputed cohort | p value |
| --- | --- | --- | --- |
| Age (years) | 64 (52-72) | 64 (52-72) | 1 |
| Heart rate (bpm) | 90 (80-104) | 89 (80-103) | 0.685 |
| Respiratory rate (bpm) | 21 (20-25) | 21 (20-25) | 0.992 |
| Temperature (℃) | 37.0 (36.5-38.0) | 37.0 (36.5-38.0) | 0.93 |
| MAP | 97 (89-105) | 97 (90-105) | 0.894 |
| SPO_2_/FiO_2_ ratio | 279 (162-331) | 279 (160-331) | 0.912 |
| WBC count (×10^9^/L) | 6.0 (4.6-8.7) | 6.0 (4.6-8.8) | 0.967 |
| NLR | 5.2 (2.8-9.9) | 5.2 (2.8-10.0) | 0.954 |
| PLT count (×10^9^/L) | 196 (143-261) | 195 (143-261) | 0.971 |
| Haemoglobin (g/L) | 127 (115-139) | 127 (116-139) | 0.866 |
| RDW (%) | 12.5 (11.9-13.4) | 12.5 (11.9-13.4) | 0.96 |
| High-sensitive C-reactive protein (mg/L) | 51.2 (11.5-105.0) | 49.8 (11.1-105.4) | 0.858 |
| Interleukin-2R (U/ml) | 793 (506-1179) | 783 (516-1181) | 0.94 |
| Interleukin-6 (pg/ml) | 21.7 (4.9-61.8) | 21.3 (5.3-57.4) | 0.877 |
| Interleukin-8 (pg/ml) | 16.2 (8.2-30.3) | 16.4 (8.6-29.5) | 0.942 |
| Tumour necrosis factor-α (pg/ml) | 8.8 (6.5-12.5) | 9.0 (6.6-12.6) | 0.764 |
| D-dimer (μg/ml) | 1.3 (0.5-3.4) | 1.3 (0.5-3.5) | 0.945 |
| Fibrinogen (g/L) | 5.0 (3.9-6.2) | 5.1 (3.9-6.1) | 0.88 |
| INR | 1.1 (1.0-1.2) | 1.1 (1.0-1.2) | 0.821 |
| Hypersensitive troponin I (pg/ml) | 8.1 (2.8-30.2) | 7.2 (2.6-23.7) | 0.359 |
| Albumin (g/L) | 34.2 (30.2-37.1) | 34.2 (30.2-37.2) | 0.933 |
| Total bilirubin (μmol/L) | 10.0 (7.2-14.2) | 10.0 (7.2-14.1) | 1 |
| Creatinine (μoml/L) | 74 (58-93) | 74 (58-93) | 0.953 |
| Urea nitrogen (mmol/L) | 5.1 (3.7-8.3) | 5.1 (3.7-8.3) | 0.982 |
| Lactate dehydrogenase (U/L) | 97 (53-203) | 318 (226-480) | 0.845 |
| Glucose (mmol/L) | 6.5 (5.5-8.6) | 6.5 (5.5-8.7) | 0.888 |

MAP= mean arterial pressure; SPO_2_/FiO_2_ ratio= ratio of pulse oxygen saturation to the fractional concentration of oxygen in inspired air; WBC= White blood cell count; NLR= Neutrophil-to-Lymphocyte ratio; RDW= Red blood cell Distribution Width; INR=International Normalized Ratio.

**Supplementary Table 3: Class-defining variables by phenotypes in sensitivity analysis excluding variables with high correlation (WBC count, Interleukin-6 and D-dimer).**

| Variables | Hypoactive phenotype  (n = 329) | Hyperactive phenotype  (n = 175) | p value |
| --- | --- | --- | --- |
| Age (years) | 59 (49-69) | 69 (62-77) | < 0.001 |
| Heart rate (bpm) | 89 (78-101) | 95 (82-108) | < 0.001 |
| Respiratory rate (bpm) | 20 (20-22) | 24 (20-32) | < 0.001 |
| Temperature (℃) | 37.0 (36.5-37.8) | 37.3 (36.5-38.0) | 0.059 |
| MAP | 96.0 (89.9-104.8) | 99.7 (89.0-106.0) | 0.288 |
| SPO_2_/FiO_2_ ratio | 297 (257-429) | 122 (90-224) | < 0.001 |
| NLR | 3.4 (2.1-5.8) | 13.5 (8.6-25.7) | < 0.001 |
| PLT count (×10^9^/L) | 213 (159-277) | 161 (120-221) | < 0.001 |
| Haemoglobin (g/L) | 126 (115-137) | 131 (116-143) | 0.024 |
| RDW (%) | 12.4 (11.9-13.2) | 13.0 (12.2-13.9) | < 0.001 |
| High-sensitive C-reactive protein (mg/L) | 27.9 (6.1-67.7) | 104.0 (63.2-167.7) | < 0.001 |
| Interleukin-2R (U/ml) | 655 (427-908) | 1263 (941-1661) | < 0.001 |
| Interleukin-8 (pg/ml) | 11.5 (6.5-19.9) | 34.1 (20.7-68.3) | < 0.001 |
| Tumour necrosis factor-α (pg/ml) | 7.9 (5.8-10.0) | 12.8 (9.0-18.8) | < 0.001 |
| Fibrinogen (g/L) | 4.9 (4.0-6.0) | 5.4 (3.2-6.4) | 0.421 |
| INR | 1.1 (1.0-1.1) | 1.2 (1.1-1.4) | < 0.001 |
| Hypersensitive troponin I (pg/ml) | 3.9 (1.9-8.5) | 45.7 (15.6-319.2) | < 0.001 |
| Albumin (g/L) | 35.9 (32.9-38.6) | 29.9 (27.1-32.4) | < 0.001 |
| Total bilirubin (μmol/L) | 8.7 (6.5-11.9) | 13.3 (9.9-19.2) | < 0.001 |
| Creatinine (μoml/L) | 66.0 (56.0-82.0) | 91.0 (73.0-123) | < 0.001 |
| Urea nitrogen (mmol/L) | 4.2 (3.2-5.5) | 9.5 (6.4-15.7) | < 0.001 |
| Lactate dehydrogenase (U/L) | 261 (204-334) | 522 (428-695) | < 0.001 |
| Glucose (mmol/L) | 6.1 (5.2-7.3) | 8.3 (6.3-12.2) | < 0.001 |

MAP= mean arterial pressure; SPO_2_/FiO_2_ ratio= ratio of pulse oxygen saturation to the fractional concentration of oxygen in inspired air; WBC= White blood cell count; NLR= Neutrophil-to-Lymphocyte ratio; RDW= Red blood cell Distribution Width; INR=International Normalized Ratio.

**Supplementary Table 4: Additional baseline characteristics and treatments of phenotypes using consensus K means clustering**

| Variables | Hypoactive phenotype  (n = 322) | Hyperactive phenotype  (n = 182) | p value |
| --- | --- | --- | --- |
| **Baseline characteristics** |  |  |  |
| Sex, n (%) |  |  | 0.026 |
| Male | 153 (47.5) | 106 (58.2) |  |
| Female | 169 (52.5) | 76 (41.8) |  |
| Fever (temperature>37.3°C), n (%) | 279 (86.6) | 160 (87.9) | 0.788 |
| Dyspnea, n (%) | 184 (57.1) | 131 (72.0) | 0.001 |
| SOFA score |  |  |  |
| Circulation | 0 (0-0) | 0 (0-0) | <0.001 |
| CNS | 0 (0-0) | 1 (0-2) | <0.001 |
| Coagulation | 0 (0-0) | 0 (0-1) | <0.001 |
| Liver | 0 (0-0) | 0 (0-0) | <0.001 |
| Renal | 0 (0-0) | 0 (0-1) | <0.001 |
| Respiratory | 2 (0-2) | 4 (3-4) | <0.001 |
| Total score | 2 (1-3) | 6 (4-8) | <0.001 |
| Vasopressor (1^st^ 24 h), n (%) | 0 (0) | 16 (8.8) | <0.001 |
| Invasive mechanical ventilation (1^st^ 24 h), n (%) | 1 (0.2) | 22 (12.1) | <0.001 |
| Non-Invasive respiratory support^*^ (1^st^ 24 h), n (%) | 7 (2.2) | 56 (30.8) | <0.001 |
| Comorbidities, n (%) |  |  |  |
| Hypertension | 109 (33.9) | 89 (48.9) | 0.001 |
| Coronary heart disease | 26 (8.1) | 29 (15.9) | 0.01 |
| Chronic heart failure | 4 (1.2) | 15 (8.2) | <0.001 |
| Diabetes | 47 (14.6) | 44 (24.2) | 0.01 |
| COPD | 11 (3.4) | 10 (5.5) | 0.374 |
| Malignancy | 11 (3.4) | 5 (2.7) | 0.883 |
| Immunodeficiency | 8 (2.5) | 2 (1.1) | 0.342 |
| **Treatments, n (%)** |  |  |  |
| Intravenous Immunoglobin | 101 (31.4) | 104 (57.1) | <0.001 |
| Corticosteroid therapy | 142 (44.1) | 143 (78.6) | <0.001 |
| High-flow nasal cannula oxygen therapy | 15 (4.7) | 31 (17.0) | <0.001 |
| Non-invasive mechanical ventilation | 23 (7.1) | 83 (45.6) | <0.001 |
| Invasive mechanical ventilation | 11 (3.4) | 108 (59.3) | <0.001 |
| ECMO | 2 (0.6) | 5 (0.5) | 0.105 |
| Renal replacement therapy | 5 (1.6) | 21 (11.5) | <0.001 |

^*^Non-Invasive respiratory support including high-flow nasal cannula oxygen therapy and non-invasive mechanical ventilation.

SOFA= Sequential Organ Failure Assessment; CNS=central nervous system; COPD= Chronic Obstructive Pulmonary Disease; ECMO= extracorporeal membrane oxygenation.

**Supplementary Table 5: Fit statistics for latent class models from one to five classes.**

| k | **AIC** | **BIC** | **Entropy^*^** | **Number of individuals per class or subphenotype** | | | | | **p value**  **k vs k-1**  **classes^#^** |
| --- | --- | --- | --- | --- | --- | --- | --- | --- | --- |
|  |  |  |  | 1 | 2 | 3 | 4 | 5 |  |
| 1 Class | 37265.5 | 37485.1 | -- | 504 | -- | -- | -- | -- |  |
| 2 Classes | 35063.6 | 35397.2 | 0.94 | 341 | 163 | -- | -- | -- | 0.0066 |
| 3 Classes | 34208.3 | 34655.9 | 0.96 | 309 | 32 | 163 | -- | -- | 0.058 |
| 4 Classes | 33596.8 | 34158.4 | 0.94 | 253 | 166 | 31 | 54 | -- | 0.41 |
| 5 Classes | 32936.6 | 33612.2 | 0.96 | 13 | 238 | 169 | 31 | 53 | 0.40 |

^*^Entropy is an index of how well the classes are separated: it ranges from zero to one and values of about 0·8 or higher are thought to be a sign of a useful mode

^#^p value represents the Vuong-Lo-Mendell-Rubin (VLMR) test, which test if k classes is a better model fit than k-1 classes.

AIC= Akaike Information Criterion; BIC=Bayesian Information Criterion.

**Supplementary Table 6: Class-defining variables of phenotypes derived using latent class analysis**

| Variables | Hypoactive phenotype  (n = 341) | Hyperactive phenotype  (n = 163) | p value |
| --- | --- | --- | --- |
| Age (years) | 59 (49-69) | 69 (62-77) | < 0.001 |
| Heart rate (bpm) | 89 (78-102) | 95 (82-108) | < 0.001 |
| Respiratory rate (bpm) | 20 (20-22) | 25 (20-32) | < 0.001 |
| Temperature (℃) | 37.0 (36.5-37.8) | 37.0 (36.5-38.0) | 0.491 |
| MAP | 97.0 (90.0-15.0) | 99.7 (88.0-105.8) | 0.490 |
| SPO_2_/FiO_2_ ratio | 297 (253-429) | 114 (90-194) | < 0.001 |
| WBC count (×10^9^/L) | 5.3 (4.1-6.6) | 9.7 (7.3-13.4) | < 0.001 |
| NLR | 3.5 (2.1-5.9) | 15.3 (9.4-26.5) | < 0.001 |
| PLT count (×10^9^/L) | 212 (158-276) | 160 (120-222) | < 0.001 |
| Haemoglobin (g/L) | 126 (116-137) | 131 (115-143) | 0.039 |
| RDW (%) | 12.4 (11.9-13.2) | 12.9 (12.2-13.9) | < 0.001 |
| High-sensitive C-reactive protein (mg/L) | 31.0 (6.9-67.7) | 107.3 (66.8-166.3) | < 0.001 |
| Interleukin-2R (U/ml) | 660 (439-927) | 1260 (940-1654) | < 0.001 |
| Interleukin-6 (pg/ml) | 12.3 (2.6-39.2) | 64.8 (30.0-162.9) | < 0.001 |
| Interleukin-8 (pg/ml) | 11.8 (6.9-20.4) | 33.5 (20.6-69.4) | < 0.001 |
| Tumour necrosis factor-α (pg/ml) | 8.1 (5.8-10.2) | 12.7 (8.8-18.9) | < 0.001 |
| D-dimer (μg/ml) | 0.7 (0.5-1.5) | 7.2 (2.1-21.0) | < 0.001 |
| Fibrinogen (g/L) | 5.0 (4.0-6.0) | 5.2 (3.1-6.3) | 0.81 |
| INR | 1.1 (1.0-1.1) | 1.2 (1.1-1.4) | < 0.001 |
| Hypersensitive troponin I (pg/ml) | 4.1 (1.9-8.7) | 49.0 (18.3-401.3) | < 0.001 |
| Albumin (g/L) | 35.9 (32.6-38.4) | 29.8 (26.8-32.4) | < 0.001 |
| Total bilirubin (μmol/L) | 9.1 (6.6-12.0) | 13.4 (9.7-19.8) | < 0.001 |
| Creatinine (μoml/L) | 66.0 (56.0-84.5) | 89.0 (72.8-120.5) | < 0.001 |
| Urea nitrogen (mmol/L) | 4.3 (3.3-5.7) | 9.6 (6.5-15.7) | < 0.001 |
| Lactate dehydrogenase (U/L) | 264 (205-339) | 536 (443-748) | < 0.001 |
| Glucose (mmol/L) | 6.1 (5.2-7.3) | 8.3 (6.5-12.4) | < 0.001 |

MAP= mean arterial pressure; SPO_2_/FiO_2_ ratio= ratio of pulse oxygen saturation to the fractional concentration of oxygen in inspired air; WBC= White blood cell count; NLR= Neutrophil-to-Lymphocyte ratio; RDW= Red blood cell Distribution Width; INR=International Normalized Ratio.

**Supplementary Table 7: The importance of top five variables from the classification tree with bagging model**

| Variables | Importance |
| --- | --- |
| NLR | 45.2 |
| SPO_2_/FiO_2_ ratio | 15.3 |
| Lactate dehydrogenase | 11.8 |
| Tumour necrosis factor-α | 6.2 |
| Urea nitrogen | 4.9 |

NLR= Neutrophil-to-Lymphocyte ratio

**Supplementary Table 8: Nested model composition and accuracy in dataset**

|  | NLR | SPO_2_/FiO_2_ ratio | LDH | TNF-α | BUN | AUC (95%CI) | AIC | p value |
| --- | --- | --- | --- | --- | --- | --- | --- | --- |
| Model 1 | Yes | No | No | No | No | 0.92 (0.89-0.94) | 371.9 | — |
| Model 2 | Yes | Yes | No | No | No | 0.94 (0.92-0.96) | 299.9 | 0.011 |
| Model 3 | Yes | Yes | Yes | No | No | 0.95 (0.94-0.97) | 273.1 | 0.038 |
| Model 4 | Yes | Yes | Yes | Yes | No | 0.97 (0.96-0.98) | 211.4 | <0.001 |
| Model 5 | Yes | Yes | Yes | Yes | Yes | 0.98 (0.97-0.99) | 208.4 | 0.336 |

NLR= Neutrophil-to-Lymphocyte ratio; SPO_2_/FiO_2_ ratio= ratio of pulse oxygen saturation to the fractional concentration of oxygen in inspired air; LDH= Lactate dehydrogenase; TNF-α: Tumour necrosis factor-α; BUN= Urea nitrogen; AUC= area under the receiver operator characteristic curve. AIC= Akaike information criterion

**Supplementary Table 9: Multivariable models assessing impact of variables on classification of pehnotypes**

|  | Three-variable model | | | Four-variable model | | |
| --- | --- | --- | --- | --- | --- | --- |
|  | OR | 95% CI | p value | OR | 95% CI | p value |
| NLR | 1.27 | 1.18-1.36 | <0.001 | 1.30 | 1.20-1.41 | <0.001 |
| SPO_2_/FiO_2_ ratio | 0.99 | 0.99-0.99 | <0.001 | 0.99 | 0.98-0.99 | <0.001 |
| LDH | 1.0048 | 1.0028-1.0068 | <0.001 | 1.0048 | 1.0026-1.0071 | <0.001 |
| TNF-α | -- | -- | -- | 1.29 | 1.19-1.4 | <0.001 |

NLR= Neutrophil-to-Lymphocyte ratio; SPO_2_/FiO_2_ ratio= ratio of pulse oxygen saturation to the fractional concentration of oxygen in inspired air; LDH= Lactate dehydrogenase; TNF-α: Tumour necrosis factor-α.

**Supplementary Figure 1: Consensus k clustering results in Tongji cohort**


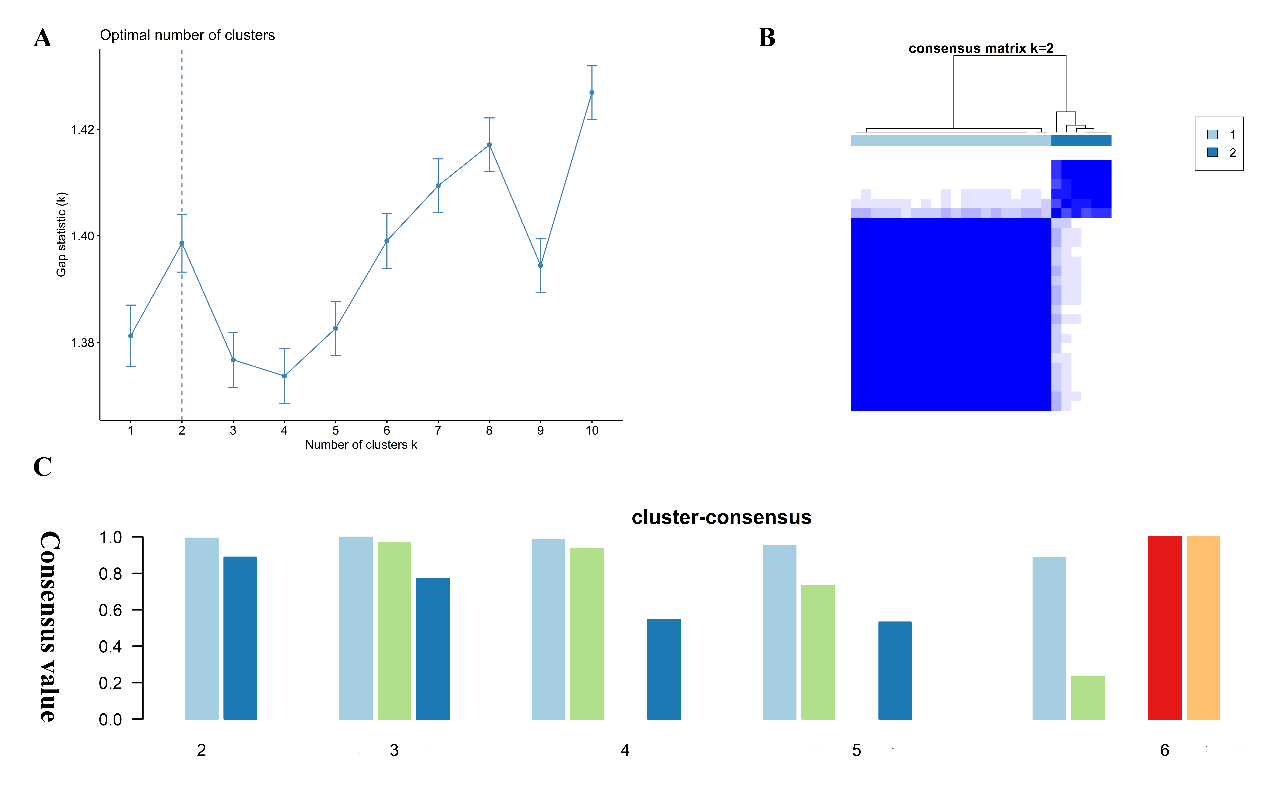


Unsupervised consensus k clustering in Tongji cohort showing the optimal number of cluster is k=2 (A) and the optimal partioning in consensus matrix for k=2 (B). Cluster consensus plot showing the mean of all pairwise consensus values between a cluster members, for k=2 to k=6 where greater values (>0.8) for all bars suggest optimal fit (C).

**Supplementary Figure 2: Heatmap of correlation between clinical variables for phenotyping**


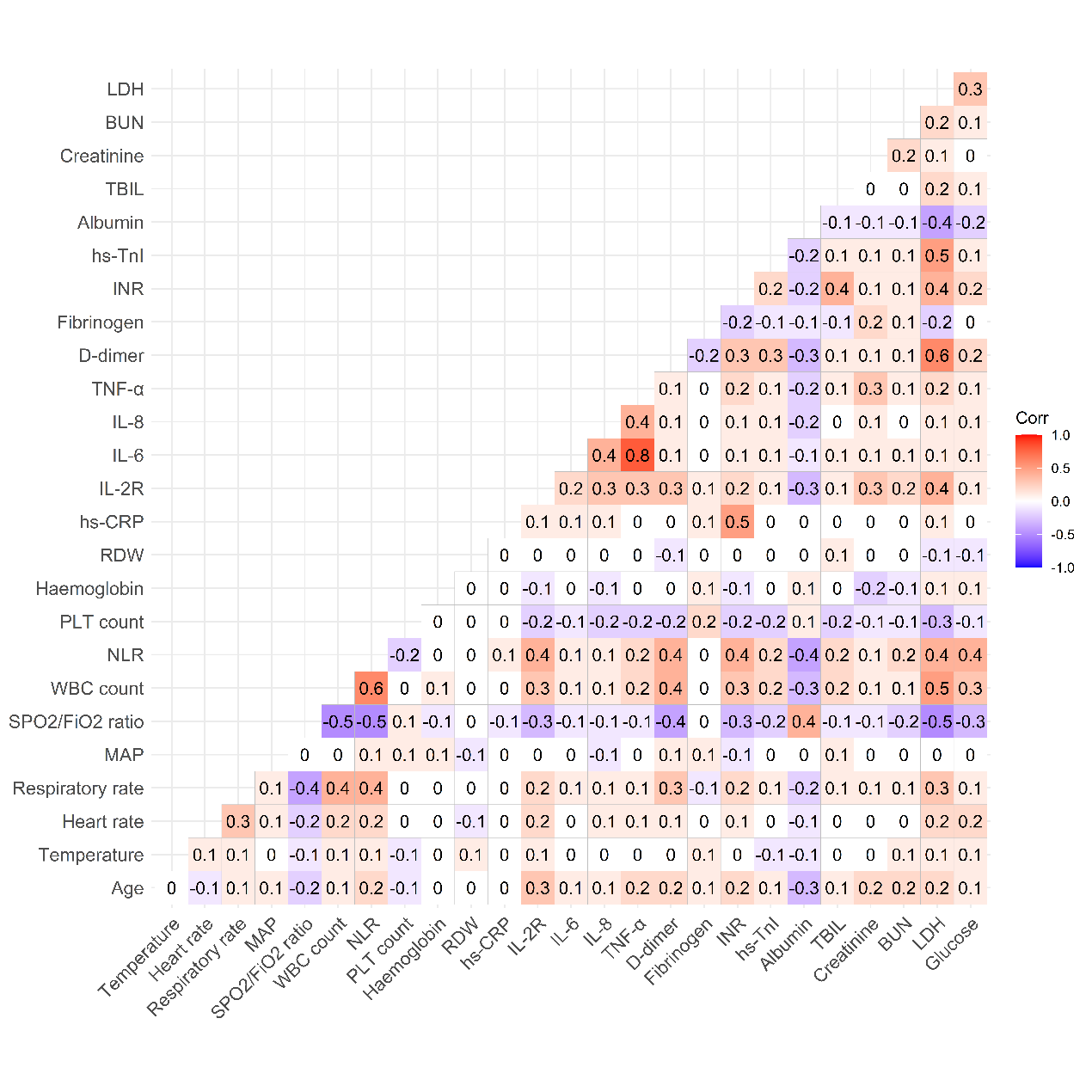


Heatmap shows greater color (red and blue) when Spearman rank order correlation coefficient is greater in positive or negative direction. Values for all correlation coefficients in the matrix were less than 0.5 except for WBC count and NLR (0.6), IL-6 and TNF-α (0.8), D-dimer and LDH (0.6).

LDH= Lactate dehydrogenase; BUN= Urea nitrogen; TBIL= Total bilirubin; hs-TnI= Hypersensitive troponin I; INR= International Normalized Ratio; TNF-α= Tumour necrosis factor-α; IL-8= Interleukin-8; IL-6= Interleukin-6; IL-2R= Interleukin-2R; hs-CRP= High-sensitive C-reactive protein; RDW= Red blood cell Distribution Width; NLR= Neutrophil-to-Lymphocyte ratio; SPO2/FiO2 ratio= ratio of pulse oxygen saturation to the fractional concentration of oxygen in inspired air; MAP= mean arterial pressure.

**Supplementary Figure 3: Difference of key class-defining variables between phenotypes**


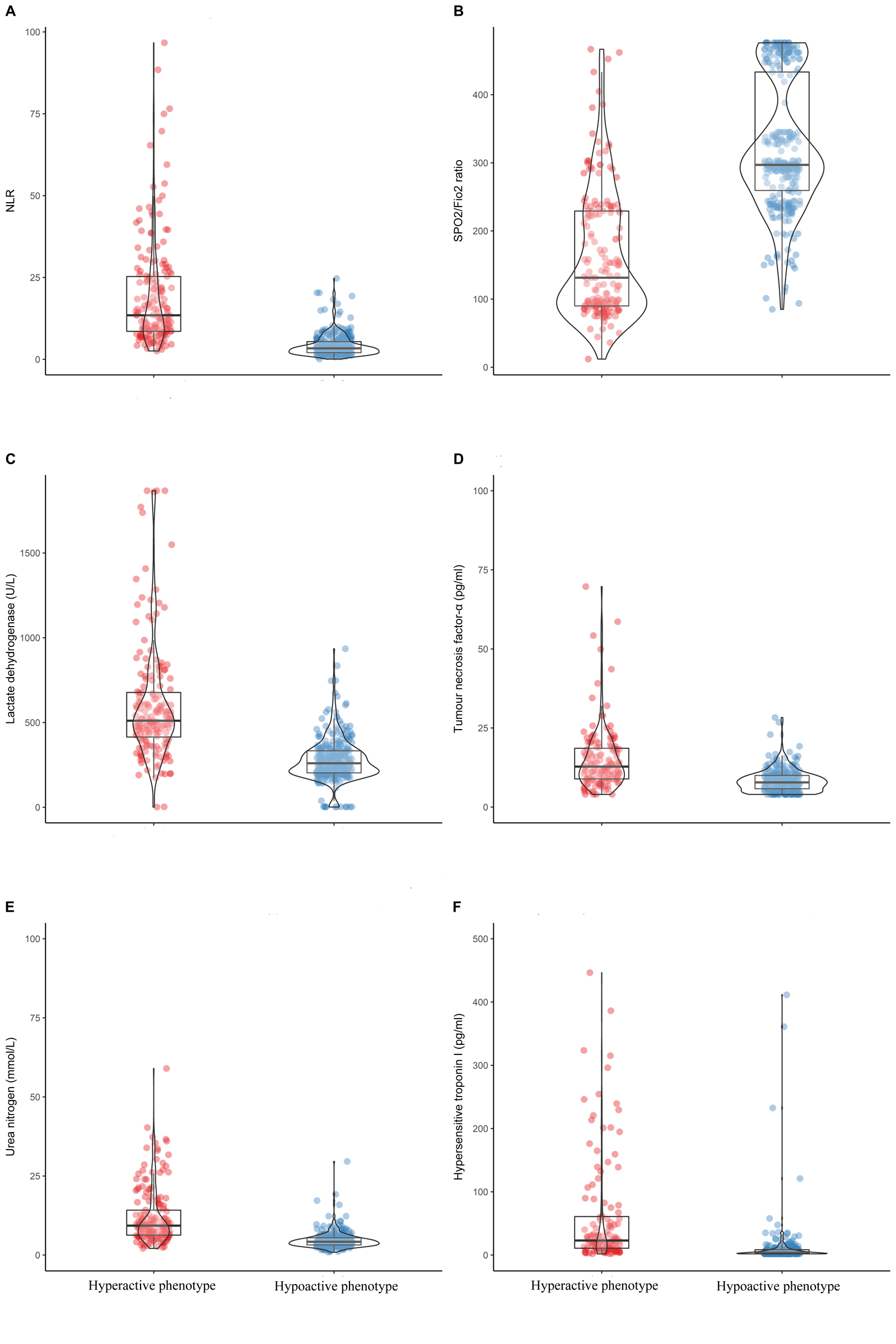


Figures shows difference in NLR (A), SPO_2_/FiO_2_ ratio (B), Lactate dehydrogenase (C) , Tumour necrosis factor-α (D), Urea nitrogen (E); High-sensitive C-reactive protein (F). Differences between phenotypes for all variables were significant.

NLR= Neutrophil-to-Lymphocyte ratio; SPO2/FiO2 ratio= ratio of pulse oxygen saturation to the fractional concentration of oxygen in inspired air.

**Supplementary Figure 4: Feature importance derived from XGBoost model**


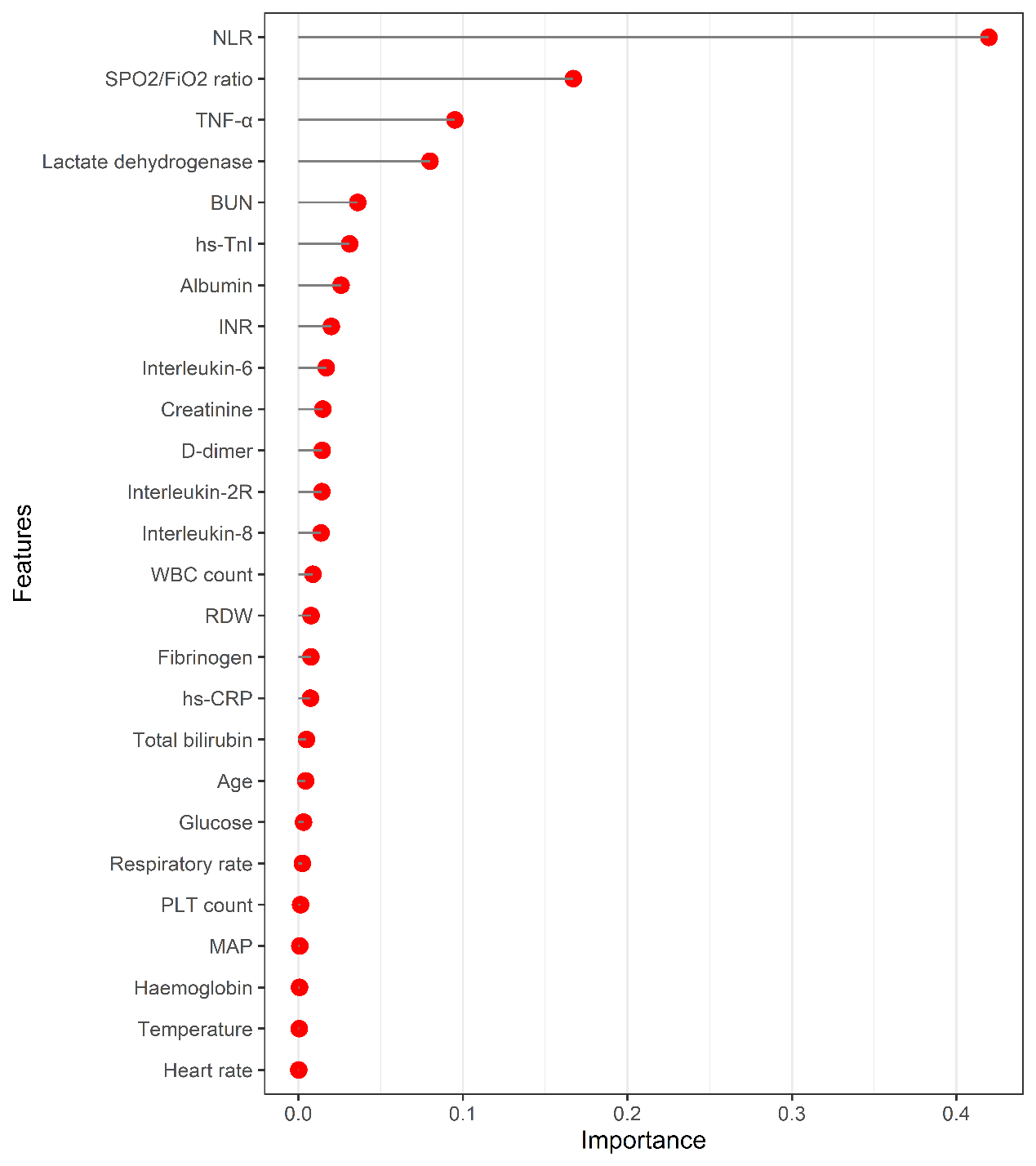


NLR= Neutrophil-to-Lymphocyte ratio; SPO2/FiO2 ratio= ratio of pulse oxygen saturation to the fractional concentration of oxygen in inspired air; TNF-α= Tumour necrosis factor-α; BUN= Urea nitrogen; hs-TnI= Hypersensitive troponin I; INR= International Normalized Ratio; RDW= Red blood cell Distribution Width; hs-CRP= High-sensitive C-reactive protein; MAP= mean arterial pressure.

**Supplementary Figure 5: Relative influence of variables derived from GBM (A) and training process of GBM (B)**


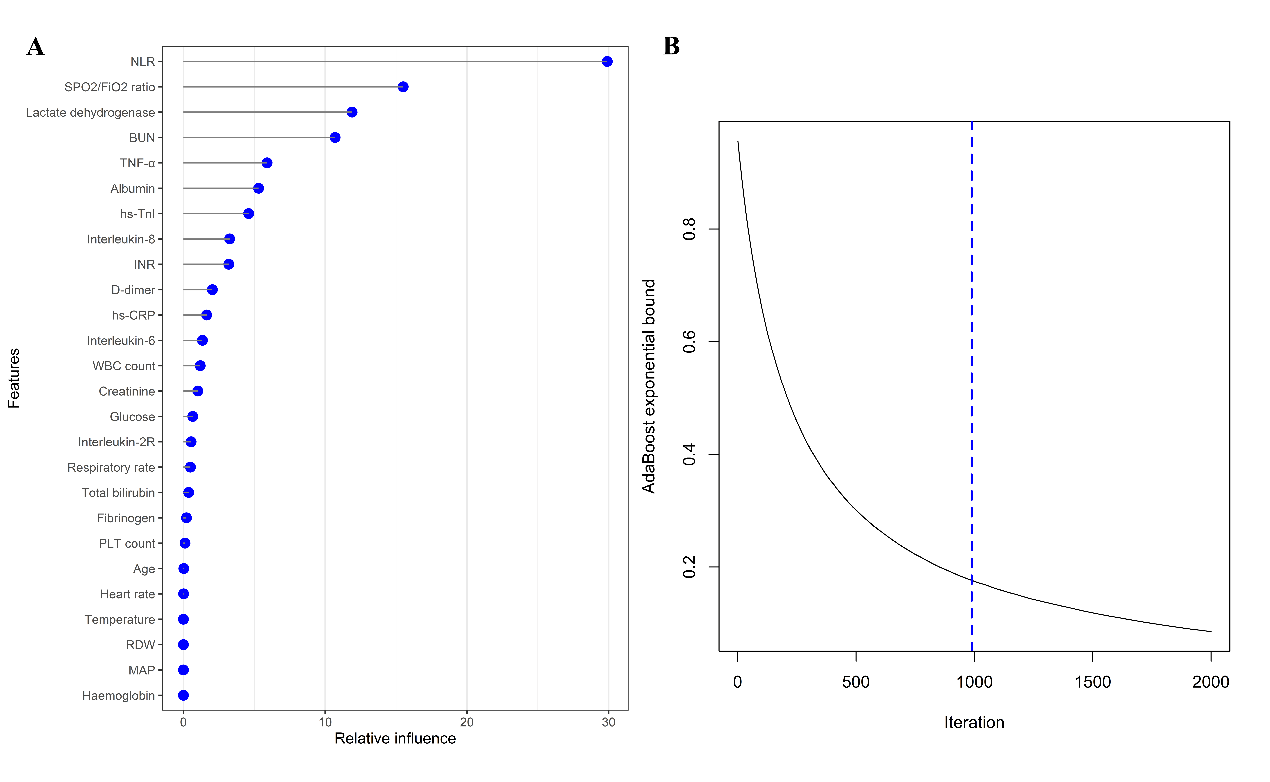


NLR= Neutrophil-to-Lymphocyte ratio; SPO2/FiO2 ratio= ratio of pulse oxygen saturation to the fractional concentration of oxygen in inspired air; BUN= Urea nitrogen; TNF-α= Tumour necrosis factor-α; hs-TnI= Hypersensitive troponin I; INR= International Normalized Ratio; hs-CRP= High-sensitive C-reactive protein; RDW= Red blood cell Distribution Width; MAP= mean arterial pressure.
